# Utility of Magnetic Resonance Imaging for Differentiating Necrotizing Fasciitis from Severe Cellulitis: A Magnetic Resonance Indicator for Necrotizing Fasciitis (MRINEC) Algorithm

**DOI:** 10.3390/jcm9093040

**Published:** 2020-09-21

**Authors:** Min-Chul Kim, Sujin Kim, Eun Been Cho, Guen Young Lee, Seong-Ho Choi, Seon Ok Kim, Jin-Won Chung

**Affiliations:** 1 Division of Infectious Diseases, Department of Internal Medicine, Chung-Ang University Hospital, Seoul 06973, Korea; Kimminchulmd@caumc.or.kr (M.-C.K.); bin0508-1@caumc.or.kr (E.B.C.); tobeservant@cau.ac.kr (S.-H.C.); 2 Department of Radiology, Chung-Ang University Hospital, Seoul 06973, Korea; netty0523@caumc.or.kr; 3 Departments of Clinical Epidemiology and Biostatistics, Asan Medical Center, University of Ulsan College of Medicine, Seoul 05505, Korea; chddk0707@naver.com

**Keywords:** necrotizing fasciitis, nonnecrotizing soft tissue infection, magnetic resonance imaging, MRINEC algorithm, LRINEC score

## Abstract

We developed a new magnetic resonance indicator for necrotizing fasciitis (MRINEC) algorithm for differentiating necrotizing fasciitis (NF) from severe cellulitis (SC). All adults with suspected NF between 2010 and 2018 in a tertiary hospital in South Korea were enrolled. Sixty-one patients were diagnosed with NF and 28 with SC. Among them, 34 with NF and 15 with SC underwent magnetic resonance imaging (MRI). The MRINEC algorithm, a two-step decision tree including T2 hyperintensity of intermuscular deep fascia and diffuse T2 hyperintensity of deep peripheral fascia, diagnosed NF with 94% sensitivity (95% confidence interval (CI), 80–99%) and 60% specificity (95% CI, 32–84%). The algorithm accurately diagnosed all 15 NF patients with a high (≥8) laboratory risk indicator for necrotizing fasciitis (LRINEC) score. Among the five patients with an intermediate (6–7) LRINEC score, sensitivity and specificity were 100% (95% CI, 78–100%) and 0% (95% CI, 0–84%), respectively. Finally, among the 29 patients with a low (≤5) LRINEC score, the algorithm had a sensitivity and specificity of 88% (95% CI, 62–98%) and 69% (95% CI, 39–91%), respectively. The MRINEC algorithm may be a useful adjuvant method for diagnosing NF, especially when NF is suspected in patients with a low LRINEC score.

## 1. Introduction

Necrotizing fasciitis (NF) is a life-threatening skin and soft tissue infection that leads to extensive tissue destruction and systemic toxicity [1]. Prompt and aggressive surgical debridement is crucial for improving survival and reducing morbidity of patients with NF [2]. However, the diagnosis of NF has remained challenging because the typical characteristics of NF such as bullae, necrosis, and hemorrhage are absent in a significant number of NF cases [1,3,4]. The laboratory risk indicator for necrotizing fasciitis (LRINEC) score, which is composed of white blood cells, hemoglobin, sodium, glucose, creatinine, and C-reactive protein, has been widely used for differentiating NF from non-necrotizing soft tissue infection (NNSTI) [5,6]. However, the LRINEC score should not be used to rule out NF since the laboratory scoring might have suboptimal sensitivity [4,7]. Therefore, further testing might be needed to resolve diagnostic uncertainty when NF is still suspected despite the LRINEC score having been applied [2].

The usefulness of magnetic resonance imaging (MRI) for diagnosing of NF has not been fully evaluated. Previous studies suggested that MRI could be helpful in discriminating NF from NNSTI [8,9,10,11,12]. However, its sensitivity and specificity for diagnosing NF are ill defined [2,5]. Importantly, the impact of MRI on the differentiation between NF and NNSTI has not been evaluated in conjunction with the LRINEC score. We therefore investigated the difference in MRI findings between patients with NF and those with NNSTI. Then, we newly developed a magnetic resonance indicator for necrotizing fasciitis (MRINEC) algorithm to differentiate NF from NNSTI using MRI findings that were suggestive of NF. Furthermore, we evaluated the diagnostic performance of the MRINEC algorithm in patients with suspected NF, according to the classification based on their LRINEC score.

## 2. Materials and Methods

### 2.1. Study Population and Definition of Necrotizing Fasciitis

Adult patients aged ≥18 years in Chung-Ang University Hospital, an 850-bed tertiary hospital in Seoul, South Korea, who were suspected of having NF between November 2010 and July 2018 were retrospectively analyzed. Patients with mild cellulitis, pyomyositis involving primarily muscles (without evidence of skin infection), and bone and joint infections were excluded. Diagnosis of NF was established when (1) direct examination during surgical exploration or histopathology of the surgical specimen revealed the infected fascia, or (2) when evidence of extensive soft tissue destruction such as hemorrhagic bullae and necrosis was found on physical examination. The remaining patients with NNSTI in whom there was no evidence of NF were diagnosed with severe cellulitis (SC). Clinical characteristics and MRI findings from patients with NF and SC were compared. The study protocol was approved by the Institutional Review Board of Chung-Ang University Hospital (1909-001-16277). The need for informed consent was waived in view of the observational nature of the study.

### 2.2. Analysis of Magnetic Resonance Imaging

MRI was performed on 1.5-T (Magnetom Avanto; Siemens, Erlangen, Germany) or 3.0-T (Achieva; Philips Medical Systems, Best, Netherlands and Skyra; Siemens, Erlangen, Germany) scanners. The MRI protocol included the axial, sagittal, and coronal imaging planes, which were obtained using T1- (T1WI) and T2- (T2WI) weighted imaging. Additionally, T1-weighted fat-suppressed fast spin-echo contrast-enhanced images were obtained in the axial, sagittal, and coronal planes. The fields of view, section thicknesses, and intersection gaps were variable for different anatomic sites of involvement. Two musculoskeletal radiologists with 9 (S.K.) and 10 (G.Y.L.) years of experience, respectively, who were blinded to the diagnoses of patients and their clinical characteristics, reviewed the MRIs independently and reached a final decision regarding the findings by consensus.

The MRIs of the enrolled patients were examined for the presence or absence of the following findings: diffuse or localized T2 hyperintensity of deep peripheral fascia, thickness of deep peripheral fascia, diffuse or localized T2 hyperintensity of intermuscular deep fascia, irregular or diffuse fascial enhancement, myositis, intermuscular or subcutaneous abscess, and subcutaneous fat edema. Deep peripheral fascia was defined as the peripheral investing layer of deep fascia that connects deep adipose tissues and muscles [10]. Intermuscular deep fascia was defined as the intermuscular layer of deep fascia that passes in between the muscles [10]. In addition, we defined “diffuse” hyperintensity as a signal intensity change of ≥75% of fasciae surrounding the muscle. An abscess was defined as a localized fluid collection with a low signal intensity on T1WI and a high signal intensity on T2WI, with peripheral rim enhancement on contrast-enhanced images.

### 2.3. Statistical Analysis

Categorical data were compared using the χ² or Fisher’s exact test, and continuous variables were analyzed using the Mann–Whitney U test. Univariable and multivariable logistic regression analyses were performed to identify predictive MRI findings for NF. The MRINEC algorithm for differentiating NF from SC was generated using a classification and regression tree analysis based on the MRI findings suggesting NF [13]. Diagnostic performance was expressed in terms of sensitivity, specificity, positive likelihood ratio, and negative likelihood ratio with a 95% confidence interval (CI). All reported *p*-values are two-sided, and *p*-values < 0.05 were considered statistically significant. Data manipulation and statistical analyses were conducted using R version 3.4.2 with the package rpart.

## 3. Results

### 3.1. Clinical Characteristics of Patients

**Figure 1:**
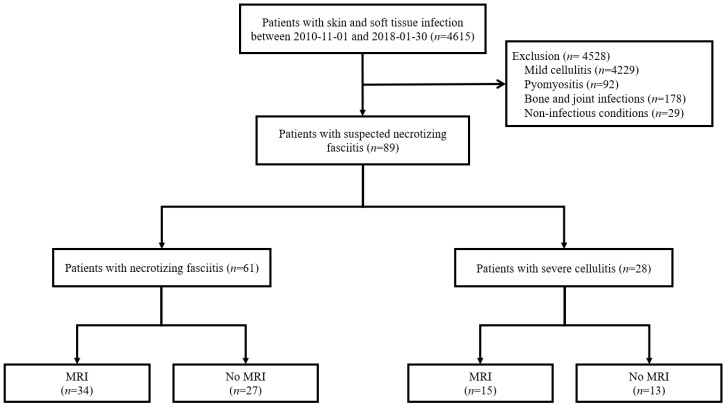
Flow diagram of the study. Abbreviations: MRI = magnetic resonance imaging.

In total, 4528 individuals were suspected of having skin and soft tissue infection from November 2010 to July 2018 in Chung-Ang University Hospital (Figure 1). Of these patients, 4229, whose diagnosis was established as mild cellulitis, were excluded. Additionally, 92 patients with pyomyositis and 178 with bone and joint infections were excluded, along with 29 who were diagnosed with non-infectious diseases. Thus, a total of 89 patients with suspected NF were finally analyzed: 61 patients were diagnosed with NF, and the remaining 28 were diagnosed with SC.

The clinical and laboratory characteristics, microbiology, and outcomes of the patients with NF and SC are shown in Table 1. Clinical characteristics such as discharge, bullae, necrosis, petechiae/hemorrhage, altered mental status, and shock were more common in patients with NF than in those with SC. White blood cell counts and C-reactive protein levels were higher, and hemoglobin and Na levels were lower, in the NF group than in the SC group. Regarding the microbiology, the causative pathogen was identified more often from blood and wound cultures in patients with NF than in those with SC. Thirty-five (57%) patients with NF underwent surgical debridement, whereas none with SC received surgical treatment. Additionally, patients with NF received longer antibiotic treatment than those with SC. The mortality rate was higher in patients with NF than in those with SC. Patients with NF had higher LRINEC scores than those with SC (*p* = 0.001). Notably, high LRINEC scores (≥8) were more common in patients with NF than in those with SC (39% vs. 7%, *p* = 0.002), although 9 (15%) and 28 (46%) patients with NF had moderate (6–7) and low (≤5) LRINEC scores, respectively. The sensitivity and specificity of high LRINEC scores (≥8) for the diagnosis of NF were 39% (95% CI, 27–53%) and 93% (95% CI, 77–99%), respectively.

### 3.2. MRI Findings of Necrotizing Fasciitis and Severe Cellulitis

**Figure 2:**
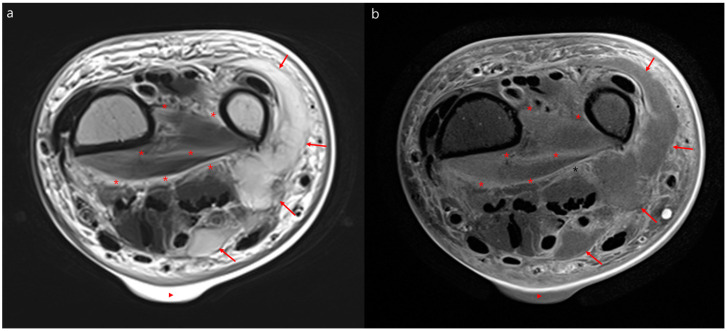
Representative magnetic resonance images of necrotizing fasciitis. Necrotizing fasciitis of the left wrist in a 71-year-old woman. Axial T2 weighted magnetic resonance image (**a**) and contrast-enhanced magnetic resonance image (**b**) showing diffuse hyperintensity with irregular enhancement of the deep peripheral fascia and intermuscular deep fascia (asterisk) of the wrist. Additionally, there is a lobulating abscess in the ulnar side of the wrist (arrows) and a skin bulla (triangle).

**Figure 3:**
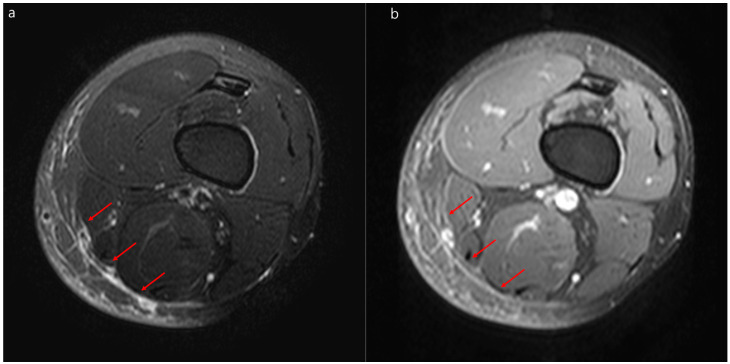
Representative magnetic resonance images of severe cellulitis. Severe cellulitis of the left thigh in a 44-year-old man. (**a**) Fat-suppressed axial T2-weighted magnetic resonance image and a contrast-enhanced magnetic resonance image (**b**) showing localized hyperintensity within the deep peripheral fascia (arrows) with enhancement in the posteromedial thigh.

Thirty-four (56%) and 15 (54%) patients with NF and SC received an MRI, respectively (*p* = 0.85) (Table 2). The intervals from admission to the receipt of MRI did not significantly differ between the NF and SC groups (*p* = 0.71). Diffuse T2 hyperintensity of deep peripheral fascia was more common in patients with NF (59%) than in those with SC (20%) (*p* = 0.01). Additionally, MRI findings of patients with NF showed T2 hyperintensity of intermuscular deep fascia (82% vs. 40%, *p* = 0.006) and diffuse T2 hyperintensity of intermuscular deep fascia (50% vs. 13%, *p* = 0.02) more frequently than those of patients with SC. Furthermore, myositis (*p* = 0.04) and intramuscular abscesses (*p* = 0.01) were more frequent in the NF group than in the SC group. However, T2 hyperintensity of deep peripheral fascia, thickness of deep peripheral fascia, localized T2 hyperintensity of intermuscular deep fascia, irregular or diffuse fascial enhancement, and subcutaneous fat edema did not significantly differ between the two groups. Representative MRIs of the patients with NF and SC are depicted in Figure 2 and Figure 3, respectively.

Results of the univariate and multivariate logistic regression analysis of MRI findings suggesting NF are shown in Table 3. The odds ratios (ORs) for diffuse T2 hyperintensity of deep peripheral fascia, T2 hyperintensity of intermuscular deep fascia, diffuse T2 hyperintensity of intermuscular deep fascia, and abscesses were 5.1 (95% CI, 1.3–20.3) (*p* = 0.02), 6.4 (95% CI, 1.7–24.6) (*p* = 0.01), 5.4 (95% CI, 1.2–25.2) (*p* = 0.03), and 9.6 (95% CI, 1.5–61.7) (*p* = 0.02), respectively. From the multivariate logistic regression analysis, diffuse T2 hyperintensity of deep peripheral fascia (OR 4.4, 95% CI, 0.9–22.8) (*p* = 0.074), diffuse T2 hyperintensity of intermuscular deep fascia (OR 5.5, 95% CI, 0.9–33.8) (*p* = 0.065), and abscesses (OR 15.1, 95% CI, 1.6–143.5) (*p* = 0.02) were suggestive of NF rather than of SC.

### 3.3. Diagnostic Performance of the MRINEC Algorithm

**Figure 4:**
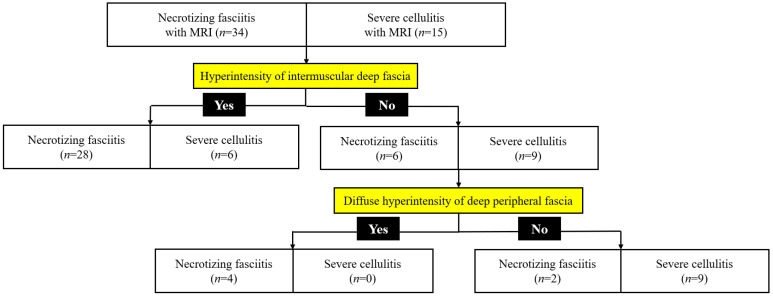
Magnetic resonance indicator for necrotizing fasciitis (MRINEC) algorithm for differentiating necrotizing fasciitis from severe cellulitis. Abbreviations: MRI = magnetic resonance imaging.

Using classification and regression tree analysis, we developed a novel MRINEC algorithm, which is a two-step decision tree, including the presence or absence of T2 hyperintensity of intermuscular deep fascia (step one), and diffuse T2 hyperintensity of deep peripheral fascia (step two) (Figure 4). The diagnostic performance of the MRINEC algorithm is shown in Table 4. Of the 49 patients with NF and SC who underwent MRIs, the overall sensitivity and specificity of the MRINEC algorithm for differentiating NF from SC were 94% (95% CI, 80–99%) and 60% (95% CI, 32–84%), respectively. The C-statistic for this algorithm was 0.79 (95% CI, 0.67–0.96). The MRINEC algorithm correctly diagnosed all 15 patients with NF with a high LRINEC score. Among the five patients with an intermediate LRINEC score, the MRINEC algorithm had a sensitivity of 100% (95% CI, 78–100%) and a specificity of 0% (95% CI, 0–84%). Furthermore, the MRINEC algorithm differentiated NF from SC with a sensitivity of 88% (95% CI, 62–98%) and a specificity of 69% (95% CI, 39–91%) among the 29 patients with low LRINEC scores.

## 4. Discussion

We evaluated the utility of MRI for the diagnosis of NF, and assessed the diagnostic performance of the MRINEC algorithm for differentiating NF from SC. The overall sensitivity and specificity of this algorithm for diagnosing NF were 94% and 60%, respectively. Notably, the MRINEC algorithm differentiated NF from SC with a sensitivity of 88% and a specificity of 69% in patients with low LRINEC scores. Thus, the MRINEC algorithm appeared to be useful for diagnosing NF, especially in cases in which the differentiation between NF and SC based on clinical and laboratory findings is difficult. Furthermore, the MRINEC algorithm may be useful for excluding a diagnosis of NF, given its high sensitivity.

Several studies have previously evaluated MRI findings suggestive of NF rather than of NNSTI [8,9,11]. Kim et al., showed that thick (≥3 mm) and extensive signal change of the deep fascia, focal or diffuse non-enhancing fascia, and the involvement of three or more compartments were more frequent in patients with NF than in those with NNSTI [8]. The MRINEC algorithm, in which T2 hyperintensity of intermuscular deep fascia and diffuse T2 hyperintensity of deep peripheral fascia were included, is consistent with the observations of the previous study. However, thickening and enhancement of the fascia were not significantly different between NF and SC in the present study. Regarding fascial enhancement, Schmid et al., reported that enhancement of deep fascia was found in all patients with NF [11], whereas the absence of enhancement was an important MRI finding, indicating fascial necrosis in Brothers et al.’s study [9]. This discrepancy could be because these earlier studies included only a limited number of NF cases, thus the MRI findings of NF might not have been fully evaluated. Furthermore, previous studies have another important limitation, in that they have not evaluated the diagnostic utility of the MRI findings in the context of the clinical judgment based on the LRINEC score. Recently, Yoon et al., reported that a new scoring system including thickening of the deep fascia ≥3 mm, multi-compartmental involvement, and LRINEC score improved sensitivity and specificity for the diagnosis of NF compared with the LRINEC score alone [14]. However, their study did not provide insights as to when MRI might be advisable, although MRI might not be feasible in a considerable number of patients with NF due to the aggressiveness of the disease. However, our MRINEC algorithm might provide additional benefits in diagnostic performance, taking into consideration the LRINEC score classifications.

Imaging studies should not delay surgical intervention in patients in whom NF is strongly suspected [1,2]. Given the rapidly deteriorating nature of NF, the LRINEC score is easily applicable and could be a useful tool in differentiating NF from NNSTI. A recent systematic review showed that an LRINEC score of ≥8 had a sensitivity and specificity of 41% and 95% for the diagnosis of NF, respectively [4]. Considering the high specificity of the LRINEC score, it might be reasonable for patients with suspected NF having high LRINEC scores to undergo surgical exploration without additional imaging evaluation. However, the LRINEC score might not be sensitive enough to diagnose NF [7,15,16,17,18,19,20,21]. Similarly, the sensitivity of LRINEC scores of ≥8 for the diagnosis of NF was low (39%) in our study. The poor sensitivity of LRINEC could be attributable to the fact that laboratory findings of patients with NF might be associated with the severity of the infection [1]. Early stages of NF might affect the low LRINEC scores [17]. In addition, immunocompromised [15] and pediatric [21] patients could have low LRINEC scores. Conversely, MRI is highly sensitive in the detection of inflammation, fluid collection, and perfusion defects in soft tissue [10,12]. However, MRI alone might overestimate the extent of deep fascial involvement, and thus the sensitivity of the MRI could exceed its specificity [11]. Therefore, the MRINEC algorithm and LRINEC scoring might be mutually complementary for differentiating NF from NNSTI.

Our study had a few limitations. First, approximately only half of enrolled patients with NF and SC received MRI in the present study. This is partially because patients with strongly suspected NF underwent surgical treatment without MRI, and further imaging evaluation was not required in patients with SC who showed a favorable response to antibiotics. Thus, there could have been a selection bias toward less aggressive cases of NF and more severe cases of SC. However, it would be more likely to lead to a bias toward the null hypothesis. Second, given that a limited number (57%) of NF patients received surgical treatment, accuracy of the diagnostic criteria for NF of this study might be questioned. There have been no specific diagnostic criteria for NF due to a wide range of clinical presentations of the disease [22]. However, inclusion of the NF cases that were not confirmed in the operating room might affect the diagnostic performance of the MRINEC algorithm. Therefore, the MRINEC algorithm needs to be further evaluated in surgically confirmed cases of NF. Third, there might be a concern about the low specificity of the MRINEC algorithm in patients with an intermediate LRINEC score. This might be because there were only two patients with SC having an intermediate LRINEC score who received MRI. Finally, the MRINEC algorithm was not validated in another cohort; therefore, further studies including larger numbers of patients with NF are needed to determine the accuracy and generalizability of the MRINEC algorithm.

In conclusion, the MRINEC algorithm may be a useful adjuvant method for diagnosing NF, especially when NF is still suspected in patients with low LRINEC scores.

## Figures and Tables

**Table 1:** Clinical and laboratory characteristics, microbiology, and outcomes of patients with necrotizing fasciitis and severe cellulitis.

Variables	Necrotizing Fasciitis (*n* = 61)	Severe Cellulitis (*n* = 28)	*p*-Value
Male sex	31 (51)	17 (61)	0.39
Age, mean years ± SD	59 ± 16	59 ± 15	0.88
Acquisition site of infection			0.03
Community-acquired infection	41 (67)	25 (89)	
Healthcare-associated infection	20 (33)	3 (11)	
Infection site			
Leg	41 (67)	20 (71)	0.69
Arm	13 (21)	4 (14)	0.43
Abdomen & pelvis	4 (7)	1 (4)	>0.99
Others	3 (5)	3 (11)	0.37
Underlying disease			
Diabetes	24 (39)	9 (32)	0.51
Trauma	22 (36)	4 (14)	0.04
Tinea pedis or onychomycosis	10 (16)	9 (32)	0.09
Malignancy	11 (18)	6 (21)	0.71
Liver cirrhosis	11 (18)	1 (4)	0.09
Peripheral artery disease	7 (12)	0	0.09
Chronic kidney disease	6 (10)	3 (11)	>0.99
Lymphedema	3 (5)	1 (4)	>0.99
Symptoms and signs			
Pain and erythema	61 (100)	28 (100)	>0.99
Fever	42 (69)	14 (50)	0.09
Discharge	27 (44)	4 (14)	0.006
Bullae	25 (41)	0	<0.001
Necrosis	17 (28)	0	0.002
Altered mental status	16 (26)	1 (4)	0.01
Shock	15 (25)	1 (4)	0.02
Petechiae/hemorrhage	11 (22)	1 (4)	0.05
Metastatic infections	6 (10)	2 (7)	>0.99
Laboratory findings			
White blood cell, mean/mm^3^ ± SD	14,000 ± 8500	10,000 ± 3800	0.05
Hemoglobin, g/dL ± SD	11.4 ± 2.6	13.5 ± 1.9	0.001
Platelet, × 10^3^/mm^3^ ± SD	225 ± 131	217 ± 87	0.90
INR ± SD	1.3 ± 0.3	1.1 ± 0.2	0.07
Bilirubin, mg/dL ± SD	1.5 ± 2.9	0.9 ± 0.8	0.56
Creatinine, mg/dL ± SD	1.2 ± 1.0	1.0 ± 0.5	0.77
Na, mEq/L ± SD	135 ± 6	137 ± 4	0.02
Glucose, mg/dL ± SD	171 ± 124	153 ± 74	0.81
C-reactive protein, mg/dL ± SD	15.7 ± 11.4	9.4 ± 9.2	0.02
Procalcitonin, ng/mL ± SD	4.8 ± 5.3	6.7 ± 6.8	0.39
Lactate, mmol/L ± SD	2.5 ± 2.2	1.4 ± 0.2	0.54
LRINEC score ± SD	6.0 ± 3.6	3.1 ± 3.4	0.001
High risk (≥8)	24 (39)	2 (7)	0.002
Moderate risk (6–7)	9 (15)	4 (14)	>0.99
Low risk (≤5)	28 (46)	22 (79)	0.004
Microbiology			
Overall culture positivity	38 (62)	5 (18)	<0.001
Blood culture positivity	18 (30)	2 (7)	0.02
Wound culture positivity	27 (44)	3 (11)	0.002
Monobacterial infection	30 (49)	5 (18)	0.005
Polybacterial infection	8 (13)	0	0.05
* Staphylococcus aureus*	11 (18)	1 (4)	0.09
Group A streptococcus	5 (8)	2 (7)	>0.99
Group B and C streptococcus	4 (7)	1 (4)	>0.99
Gram negative bacteria	18 (30)	1 (4)	0.006
Anaerobes	0	0	
Surgical debridement	35 (57)	0	<0.001
Amputation	4 (11)	0	0.30
Interval between admission and surgery, mean days ± SD	6 ± 8	NA	NA
Second look operation	18 (51)	0	0.001
Antibiotics			
Total days of antibiotics use ± SD	35 ± 25	20 ± 12	0.007
Days of intravenous antibiotics use ± SD	23 ± 19	8 ± 6	<0.001
Days of oral antibiotics use ± SD	20 ± 16	13 ± 9	0.03
Clinical outcomes			
Intensive care unit admission	16 (26)	1 (4)	0.01
Mechanical ventilator	10 (16)	0	0.03
Relapse within 1 month	10 (16)	1 (4)	0.16
In hospital mortality	14 (23)	0	0.004

NOTE. Data are presented as the number (%) of patients unless otherwise indicated. Abbreviations: INR = international normalized ratio; LRINEC = laboratory risk indicator for necrotizing fasciitis; NA = not available; SD = standard deviation.

**Table 2:** Magnetic resonance findings of necrotizing fasciitis and severe cellulitis.

Variables	Necrotizing Fasciitis (*n* = 34)	Severe Cellulitis (*n* = 15)	*p*-Value
Interval from admission to MRI, mean days ± SD	4 ± 7	4 ± 5	0.71
T2 hyperintensity of deep peripheral fascia	31 (91)	12 (80)	0.35
Diffuse	20 (59)	3 (20)	0.01
Localized	11 (32)	9 (60)	0.07
Thickness of superficial fascia, mm ± SD	6.9 ± 4.8	5.5 ± 3.0	0.52
T2 hyperintensity of intermuscular deep fascia	28 (82)	6 (40)	0.006
Diffuse	17 (50)	2 (13)	0.02
Localized	11 (32)	4 (27)	0.75
Fascial enhancement	28/31 ^a^ (90)	12/14 ^b^ (86)	0.64
Irregular	6/31 ^a^ (19)	0	0.16
Diffuse	22/31 ^a^ (71)	12/14 ^b^ (86)	0.46
Myositis	24 (71)	6 (40)	0.04
Abscess	17 (50)	1 (7)	0.004
Intermuscular abscess	11 (32)	0	0.01
Subcutaneous	6 (18)	1 (7)	0.41
Subcutaneous fat edema	30 (88)	14 (93)	>0.99

NOTE. Data are presented as the number (%) of patients unless otherwise indicated. Abbreviations: MRI = magnetic resonance imaging; SD = standard deviation. ^a^ Thirty-one patients with necrotizing fasciitis underwent enhanced magnetic resonance imaging. ^b^ Fourteen patients with severe cellulitis received enhanced magnetic resonance imaging.

**Table 3:** Multivariable logistic regression analysis of predictive magnetic resonance findings for necrotizing fasciitis.

Univariable Logistic Regression Analysis.	Odds Ratio (95% CI)	*p*-Value	Multivariable Logistic Regression Analysis	Odds Ratio (95% CI)	*p*-Value
Diffuse T2 hyperintensity of deep peripheral fascia	5.1 (1.3–20.3)	0.02	Diffuse T2 hyperintensity of deep peripheral fascia	4.4 (0.9–22.8)	0.074
T2 hyperintensity of intermuscular deep fascia	6.4 (1.7–24.6)	0.01			
Diffuse T2 hyperintensity of intermuscular deep fascia	5.4 (1.2–25.2)	0.03	Diffuse T2 hyperintensity of intermuscular deep fascia	5.5 (0.9–33.8)	0.065
Abscess	9.6 (1.5–61.7)	0.02	Abscess	15.1 (1.6–143.5)	0.02
Intermuscular abscess	15.2 (0.7–313.7)	0.078			

Abbreviations: CI = confidence interval.

**Table 4:** Diagnostic performance of the magnetic resonance indicator for necrotizing fasciitis (MRINEC) algorithm.

	NF by MRINEC/Patients with NF (*n* = 34)	SC by MRINEC/Patients with SC (*n* = 15)	Sensitivity, % (95% CI)	Specificity, % (95% CI)	PLR (95% CI)	NLR (95% CI)
**Total** (*n* = 49)	32/34	9/15	94 (80–99)	60 (32–84)	2.4 (1.3–4.4)	0.1 (0.02–0.4)
High risk, LRINEC score ≥8 (*n* = 15)	15/15	0	100 (78–100)	NA	1.0	NA
Intermediate risk, LRINEC score 6–7 (*n* = 5)	3/3	0/2	100 (29–100)	0 (0–84)	1.0	NA
Low risk, LRINEC score ≤5 (*n* = 29)	14/16	9/13	88 (62–98)	69 (39–91)	2.8 (1.2–6.6)	0.2 (0.05–0.7)

Abbreviations: CI = confidence interval; MRINEC = magnetic resonance indicator for necrotizing fasciitis; NF = necrotizing fasciitis; NLR = negative likelihood ratio; PLR = positive likelihood ratio; SC = severe cellulitis.
